# Genetic determinants of food preferences: a systematic review of observational studies

**DOI:** 10.1186/s40795-024-00828-y

**Published:** 2024-02-02

**Authors:** Jalal Hejazi, Roksaneh Amiri, Shadi Nozarian, Ronia Tavasolian, Mehran Rahimlou

**Affiliations:** 1 Department of Nutrition, School of Medicine, Zanjan University of Medical Sciences, Zanjan, Iran; 2 Department of Student Research Committee, School of Medicine, Urmia University of Medical Sciences, Urmia, Iran; 3 Department of Nutrition, Ahvaz Jondishapur University of Medical Sciences, Ahvaz, Iran; 4 Department of Nutrition, School of Public Health, Zanjan University of Medical Sciences, Zanjan, Iran

**Keywords:** Food preference, Genetic, Sweet, Genotype, Adults, Food choices

## Abstract

**Background:**

Over the last decade, the results of several studies have indicated that adults' food preferences, consumption, and dietary choices vary depending on their genotype characteristics. However, the results of studies related to genes and polymorphisms involved in this phenomenon are contradictory. This study is a systematic review designed to evaluate the genetic determinants of food preferences.

**Methods:**

This study was conducted following the guidelines of the Preferred Reporting Items for Systematic Reviews and Meta-Analyses (PRISMA). Searches were conducted to identify articles testing the impact of genotypes on food choices, preferences, and intake in healthy adults. The search included all relevant keywords, and studies published between 1/1/1994 and October 2022 were considered. We assessed the quality of included studies and evaluated the risk of bias using the Newcastle–Ottawa Scale (NOS) for observational studies.

**Results:**

A total of 8,510 records were identified through our search method, and finally, 50 studies were included in this study. The majority of the studies evaluated the association of genetic variants with preferences for macronutrients, sweet, bitter, and fatty foods. The results of our study suggest a significant correlation between TAS2R38 variants (rs713598, rs1726866, rs10246939) and bitter and sweet taste preferences. Additionally, we found a considerable association between the T102C polymorphism of the 5-HT2A receptor gene and a higher intake of protein, and rs1761667 (CD36) was associated with fat preference.

**Conclusion:**

In conclusion, this study revealed a significant association between certain genetic variants and food preferences among adults.

**Supplementary Information:**

The online version contains supplementary material available at 10.1186/s40795-024-00828-y.

## Background

Food choice is a complex process that can impact various aspects of health, including our body composition. Numerous factors may influence our food choices, including the taste of food, intrapersonal determinants such as perceptions, beliefs, attitudes, and motivations, interpersonal determinants (such as significant others), social, cultural, and environmental determinants (such as food availability, the market, etc.), and economic determinants [1, 2]. Taste is one of the most crucial determinants of food choices; however, perceptions and preferences for different tastes vary widely among individuals [3]. Genetic polymorphisms in genes involved in taste perception, at least in part, can explain these interindividual variations [3, 4].

Food preferences are influenced by a multitude of environmental, cultural, nutritional, and genetic factors [5, 6]. The initial indications of the genetic impact on food preferences were observed through investigations involving families and twins [7, 8]. In recent decades, significant progress in molecular genetics has transformed the understanding of individual variations across various aspects of human behavior. These breakthroughs empower researchers with the means to conduct extensive genetic association studies, enabling a deeper exploration of the involvement of particular gene loci in sensory perceptions, food preferences, liking or disliking, as well as habits related to food intake on a larger scale [9, 10]. here is a relatively large number of studies that have investigated the association between single nucleotide polymorphisms (SNPs) in different genes [11] especially taste receptors for sweet and umami (T1R) genes [12]. However, the association between food preference and genes seems to be much more complicated, and probably much more genes are involved in this regard [13–15].

Food hedonic questionnaires are often used to assess food preferences. These questionnaires gauge how much a person "likes" or "wants" a particular product [16, 17]. Previous studies have reported that some of the significant food preferences include sweet and savory snacks, high-protein foods, and fatty foods. Additionally, it has been noted that food preferences differ across gender and age groups [18–20].

Recent advances in genetics and the development of genome-wide association studies (GWAS) have brought a unique opportunity to gain a more holistic view of the impact of genes on food preferences [21]. Therefore, this study was designed as a systematic review to evaluate the genetic aspects of food preference in human studies among adults.

## Methods

### Study design and search strategy

The study was conducted following the guidelines outlined in the Preferred Reporting Items for Systematic Reviews and Meta-Analyses (PRISMA) [22]. The PRISMA checklist can be found in Supplementary Table S1. The study protocol has been registered with PROSPERO (CRD42022352920). In August 2022, searches were conducted on seven electronic databases: PubMed, Scopus, Cochrane Library, Web of Science, ClinicalTrials.gov, Embase, and OpenGrey. The searches involved a combination of key terms related to genetics and food preferences (see Supplementary Methods 1). There were no language restrictions in our search. Additionally, for the grey literature search, we assessed conference papers. If a study met the necessary criteria, we contacted the corresponding author to obtain the full text or required information. Our search also included review publications, editorials, letters to editors, conference papers, and the references of all the included studies. Ethical approval from the local institutional ethics committee was not required for this study, as we used previously published data.

### Eligibility criteria

Studies were included in this analysis if they were conducted among human subjects with adult participants (> 18 years). To be eligible for inclusion, studies needed to incorporate both food preferences and genotypes. Studies that solely assessed preferences for basic tastes (using glucose or salt solutions and not food) or alcoholic drinks (without food) were excluded. Studies that assessed people's food intake or eating habits without referencing food preferences were also not included. Additionally, studies that only explored the relationship between heredity and food preferences without examining specific genes were excluded from the analysis.

### Study selection

Initially, researchers conducted the search process in electronic databases. In the second stage, two researchers (MR and JH) independently performed the initial screening of the studies entered into the Endnote software. Finally, in the subsequent stage, a secondary screening was conducted by examining the full text of the articles based on the inclusion and exclusion criteria, and the final articles were selected for inclusion in this systematic review. During this stage, researchers primarily used article titles and abstracts as selection criteria. Any inconsistencies between the two researchers in the selection of studies were resolved through re-examination by each of them and consultation with a third person (RA).

### Data extraction

Two researchers, MR and JH, independently extracted the necessary information, including (a) study-related variables (first author's name, publication year, sample size, study design, the presence of a control group, and its general description), evaluated genes, the method for assessing food preferences, and the main results.

### Quality assessment

Using the Newcastle–Ottawa Scale (NOS) for observational studies, we assessed the risk of bias and rated the quality of the included research [23]. The scale employs a "star system" with a maximum of ten points, assigning points for factors such as study group selection, group comparability, exposure measurement, and result measurement. A study with five or more points was considered to be of high grade [24].

### Statistical analysis

Due to substantial variations in the outcomes under investigation, a meta-analysis was not feasible. Instead, this study was conducted as a systematic review.

## Results

### Characteristics of included studies

**Fig. 1:**
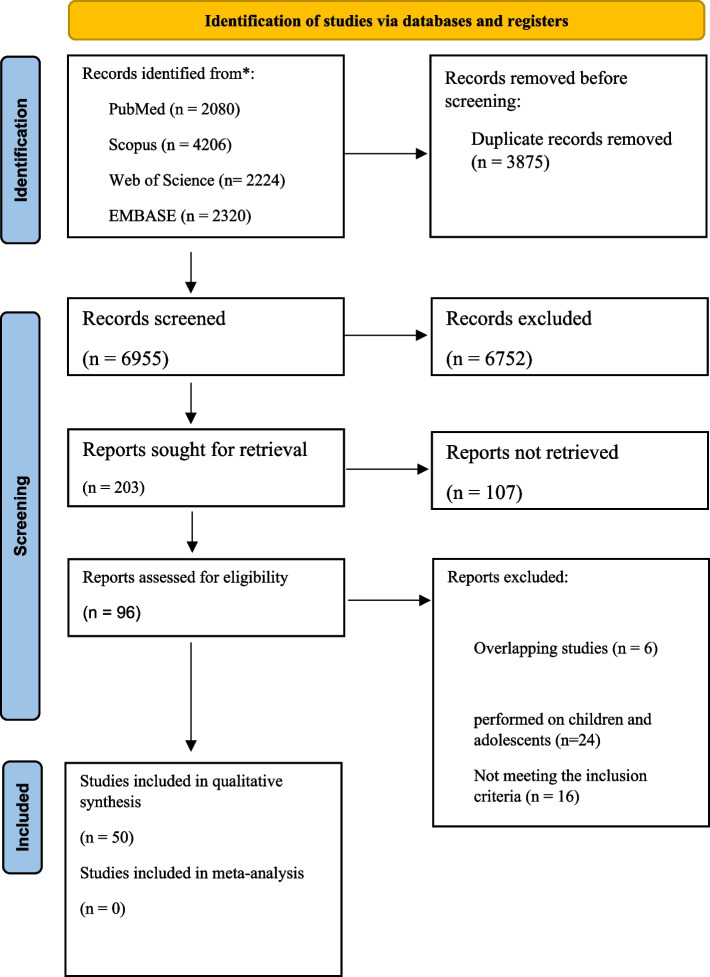
Preferred reporting items for systematic reviews and meta-analyses flow diagram of search process

Figure 1 displays a PRISMA flow diagram summarizing the inclusion procedure. In total, 8,510 items were identified using our systematic searching method. Of these, 2,634 were eliminated as duplicate records. The titles and abstracts of the remaining 5,876 records were screened, resulting in 203 articles being included in the next step. In the second phase of screening and after reviewing the full text of the studies, 50 studies met the necessary conditions for inclusion in this systematic review [9, 14, 21, 25–71]. Table 1 provides a summary of the features of the studies that were part of this systematic review. Participants were from different geographic regions, including: Japan [14, 25, 27, 39, 55, 64, 65, 68, 71],USA [32, 33, 42, 44, 60], Brazil [26], Finland [9, 38, 41], Czech Republic [28, 29, 50], Netherlands [30], Malaysia [31, 52], Caucasus [34], Israel [36], Turkey [37], Italy [21, 40, 43, 48, 53, 65], Australia [45, 51], UK [46, 49, 56, 57, 62, 67], India [47], Sweden [35, 54], Korea [58, 59], Hungary [61], Pakistan [63], and Spain[66]. The age range of the people examined in the studies was between 18 and 70 years. Most of the studies were conducted on both men and women. Also, most of the studies were conducted on healthy people. However, in four studies, patients with migraine [9], gestational diabetes mellitus (GDM) [50], obese [28, 29] and metabolic syndrome [66] were evaluated. In most studies, standard food questionnaires such as food frequency questionnaire (FFQ) or 24-h food recall were used to evaluate food preferences. However, in some studies, specific questionnaires of food preferences or interviews were applied. Also in term of evaluated genes, a wide variety of genes and SNPs have been evaluated in these studies.

**Table 1:** Characteristics of the studies

Study	Study Design	Location/Ethnicity	Sample (M, F)	Age (Years)	Health status	Food preference Assessment tools	Associated Trait	Gene	NOS Score
Ozawa, 2002 [25]	Cross-sectional	Japan/Japanese	99(72m,27f)	18–28 years (mean 20.4 years)	healthy	FFQ	preference and intake of cow’s milk	HLA genes (DRB1, DQA1 and DQB1)	5
Mennella,2005 [69]	Cross-sectional	US/Different ethnicities	114	35.3 ± 0.6 years	healthy	interview	sweet preferences	TAS2R38	4
Prado-Lima, 2006 [26]	Cross-sectional	Brazil	240	-	healthy	24-h recall and FFQ	protein intake and animal food consumption	serotonin receptor 5-HT2A	4
Keskitalo,2007 [9]	Cross-sectional	Finland	146 (46m, 100f)	49.0 ± 14.8 y	migraine	Questionnaire	sweet foods	-	7
Mizuta, 2008 [27]	Cross-sectional	Japan	2,620	64.33	healthy	Questionnaire	Sweet food	*LEP* *LEPR*	5
Bienertova-Vasku, 2008 [28]	case–control	Czech Republic	185	50.0	Obese and normal weight	A specific food records Questionnaire	percentage of protein, carbohydrates or fat or fiber in food	leptin, leptin receptor, adiponectin, proopiomelanocortin and ghrelin genes	8
Bauer, 2009 [30]	Cross-sectional	Netherlands / Dutch	1700 women	57.22 ± 6.06	healthy	semi quantitative, FFQ	Protein intake, Fat intake, Carbohydrate intake	FTO, MC4R, KCTD15, TMEM18	9
Bienertová-Vašků, 2010 [29]	Cross-sectional	Czech/ Caucasian	409	18.1–73.9	Obese and normal weight	7-day food records	percentage of protein, carbohydrates or fat	leptin (LEP), LEP receptor (LEPR), adiponectin	3
Ooi, 2010 [31]	Cross-sectional	Malaysia/Malays, Chinese and Indians	215 (100 males, 115 females)	21.3 ± 10.4	healthy	Questionnaire	like or dislike of list of Asian vegetables, soy products and 37 sweet or fat foods	TAS2R38	3
Hayes, 2011 [32]	Cross-sectional	USA/ Mostly European ancestry (85%)	96 (76% female)	40.9 years ± 12.2	healthy	general Labeled Magnitude Scale (gLMS)	Like/dislike of grapefruit juice and instant espresso	TAS2R16 TAS2R3 TAS2R4	5
Keller, 2012 [33]	Cross-sectional	USA/ African-American	317(137 male and 180 female)	35.5 ± 11.3	healthy	self-reported questionnaire	Acceptance and liking score of different fat containing food	CD36	5
Pirastu, 2012 [34]	Cross-sectional	Caucasus (Georgia, Azerbaijan, Uzbekistan, Kazakhstan and Tajikistan)	478 (199 males and 279 females)	36.2 ± 17.4	healthy	Food preferences questionnaire	Liking ratings of 20 common foods	BDNF/BDNFOS CD36 GNB3	6
Eriksson, 2012 [36]	Cross-sectional	Israel	14,604		Healthy	interview	cilantro preference	GWAS	7
Brunkwall, 2013 [35]	Cross-sectional	Malmo in Sweden	22,799 (8,797 men and 14,002 women)	-	healthy	7-day menu book; 2) 168-item questionnaire	dietary intake from 27 food groups	FTO	9
ERGÜN, 2013 [37]	Cross-sectional	Turkey	178 (59 men and 119 women)	28.72 ± 9.35	Healthy	3-day food records	food choices	hTAS2R38	3
Laaksone, 2013 [70]	Cross-sectional	Finland	41 (32 females and 9 males)	20–60	healthy	a nine-point balanced hedonic scale	The liking of odor, appearance, and flavor of Wild bilberries and Wild crowberries’ juice, extract and juice + extract	hTAS2R38	3
Sasaki, 2013 [71]	Cross-sectional	Japan	52 females	21.6 (21–22)	healthy	FFQ	sweet preference	ACE (angiotensin-converting enzyme), ADRB3 (adrenergic b3 receptor) and AGT(angiotensinogen)	5
Wakai, 2013 [39]	Cross-sectional	Japan	5,430 (52.3% women)	54.4 ± 10.9	healthy	a self-administered questionnaire	Confectionery-intake score	GWAS ADIPOQ (adiponectin encoding gene)	6
Pirastu, 2014 [40]	Cross-sectional	Italy/ Europe and Central Asia	4066 (2389 female)	49.3	healthy	coffee-liking on a 9-point scale	Coffee Liking	24 TAS2R genes	7
Törnwall, 2014 [41]	Cross-sectional	Finland	331 twins (146 men and 185 women)	22 (21–25)	healthy	The Specific Food (SF) questionnaire	liking responses to food names representing sour, umami, and spicy flavor qualities	GWAS TAS1R1 PKD1L3	7
Hayes, 2015 [42]	Cross-sectional	USA/ mostly Caucasians (72%)	246 (99 men)	18–45	healthy	The Specific Food (SF) questionnaire	liking of “unsweetened grapefruit juice.”	TAS2R19 TAS2R31	3
Robino, 2015 [43]	Cross-sectional	Italy/Caucasian	647(285 males and 362 females)	44.9 ± 12.4	healthy	a 45-item food liking questionnaire	sweet foods	TAS1R2 GLUT2	8
Wallace, 2015 [44]	Cross-sectional	USA	61 (34 female)	18–33	healthy	Pictures of eighty food items	food desirability and self-rated “healthy” and “unhealthy” food perceptions	Dopamine-related catechol-Omethyltransferase (COMT)	6
Jayewardene, 2016 [45]	Cross-sectional	Australia	56 (28 male and 28 female)	24.9 ± 3.3	healthy	self-reported habitual diet questionnaires	self-reported fat preference	CD36	6
Shen, 2016 [46]	Cross-sectional	UK/ mostly Caucasian (75%)	136 (95 females and 41 males)	18–55	healthy	FFQ	liking of four vegetables)	hTAS2R38 CA6	5
Pirastu, 2016 [21]	Cross-sectional	Italy	3856 (1591 men)	49.5	healthy	liking for each food on a scale ranging from 1 to 9	Liking/disliking for 20 foods	GWAS	7
Deshaware, 2017 [47]	Cross-sectional	India	393 (212 males, 181 females)	35.9 ± 12.0	healthy	FFQ And a 59- item food list	preferences of fruits, vegetables and dairy products	TAS2R38	5
Risso, 2017 [48]	Cross-sectional	Italy /(mostly Italians (*n* = 111)	183 (81 females	42.71 ± 15.89	healthy	questionnaire	a single 1-to-6 liking + consumption score	TAS2R1 TAS2R4	5
Shen, 2017 [49]	Cross-sectional	UK/ mostly Caucasian (75%)	136 (95 females and 41 males)	18–55	healthy	FFQ, 3-day food diary	liking of ice cream and dietary fat intake	CD36 CA6	5
Bartáková, 2018 [50]	Cross-sectional	Czech Republic/ Caucasian	363 pregnant women	Cases (33 [29–36]) Controls (32 [29–35])	GDM (*n* = 293) Healthy (*n* = 70)	food frequency questionnaire	daily frequency of intake of eight categories of foods	TAS1R2 TAS2R7	3
Han, 2018 [51]	Cross-sectional	Australia	30	27.4 (20–37)	healthy	access to a variety of foods	sweet foods	TAS1R1 TAS1R3	3
Lek, 2018 [52]	Cross-sectional	Malaysia/ Chinese(308), Indians (86)	394 (161 males, 233 females)	20.9 ± 0.12	healthy	7-point hedonic scale	The preference to high-fat Malaysian foods	DRD2	5
Perna, 2018 [53]	Cross-sectional	Italy	118 (24 men and 94 women)	45.28 ± 12.84	healthy	A food preference questionnaire	Preference of 30 food items	TAS2R38	5
Eriksson, 2019 [54]	Cross-sectional	Sweden/ European ancestry	127(60 male and 167 female)	18–23	healthy	habitual diet intake and food item preference	Score for food preference	TAS1R1, TAS1R2, TAS1R3, TAS2R16, TAS2R38, TAS2R50, SLC2A2, SLC2A4	5
Watanabe, 2019 [55]	Cross-sectional	Japan/Japanese	53 (25 men and 28 women)	24.3 ± 1.5	healthy	self-reported questionnaire	rate the degree of bitter, sour, salty, sweet or greasy (high-fat) food preference	Beta3-adrenergic receptor (ADRB3)	6
Hwang, 2019 [56]	Cross-sectional	UK/ White	174,424	56.41 ± 7.9	healthy	24-h dietary recall questionnaires	Intake of total sugars Intake of sweets	FTO TAS1R2 TAS1R3	8
Pilic, 2020 [57]	Cross-sectional	UK/ mostly Caucasians (85%)	95(32 males and 63 females)	27.6 (18–35)	healthy	two 24-h dietary recalls	Dietary salt intake and preference of salty foods	SCNN1B TRPV1	6
Kawafune, 2020 [14]	Cross-sectional	Japan	12,312 (female = 5759)	> 18	healthy	internet-based questionnaires	The taste preference for sweetness	Aldehyde dehydrogenase (ALDH2) gene	8
Park, 2020 [58]	Cross-sectional	Korea	8,842 (4,183 men and 4,659 women)	40–69 years	healthy	Questions like: “Do you like sweet foods?”, “Do you like salty foods? Etc	Preferences for each taste	TAS1R2 SLC2A5	8
Choi, 2021 [59]	Cross-sectional	Republic of Korea/ Korean	6620 (3194 males and 3425 females)	51.4	healthy	FFQ	Preference to carbohydrate- and fat-rich foods	CD36	9
Cornelis, 2021 [60]	Cross-sectional	USA: Nurses’ Health Study (NHS)	86006	66.44		Food Preferences Questionnaire	liking or consumption of coffee, tea and other bitter tasting foods	GWAS	9
Di´oszegi, 2021 [61]	Cross-sectional	Hungary/ Hungarian, Roma	Hungarian (*n* = 410) Roma (*n* = 387)	Hungarian: 44.3 ± 12.3 Roma: 42.8 ± 12.1	healthy	food preference questionnaire	Rating of sweet-, fatty-, salty- and bitter-tasting food items	TAS1R3 CD36	9
Graham, 2021 [62]	Cross-sectional	UK/ Caucasian	88 (49 females and 39 males)	35 ± 1	healthy	a semi-quantitative food frequency questionnaire	total carbohydrate, total fat	CD36 TAS2R38	5
Rana, 2021 [63]	Cross-sectional	Pakistan	578 (321 male and 257 female)	29.79 (20–63)	healthy	A self-reported questionnaire	Tendency toward fat-dense food	MC4R BDNF FTO TMEM18 NEGR1	5
Suzuki, 2021 [64]	Cross-sectional	Japan	14,079 (55% women)	54.8 ± 9.4	healthy	a semi-quantitative FFQ	preference for a Japanese dietary pattern	GWAS	9
Concas, 2022 [65]	Cross-sectional	Italy	1124 (60.7% women)	56.1 ± 16.5	healthy	a questionnaire	Liking of different foods and beverages	CAV1 (caveolin 1) GWAS	9
Fernández-Carrión, 2022 [66]	Cross-sectional	Spain/ Caucasian	425 (183 man and 242 women)	65.2 ± 4.7	subjects with metabolic syndrome	A food liking questionnaire	The preference for sugary foods	GWAS PTPRN2 (Protein Tyrosine Phosphatase Receptor Type N2)	8
May-Wilson, 2022 [67]	Cross-sectional	UK/ European descent	161,625	37–73	healthy	an online questionnaire comprising 152 items	Food-liking phenotypes	GWAS	9
Narita, 2022 [68]	Cross-sectional	Japan/ Japanese	52 (26 Male and 26 female)	23.4 ± 3.1	healthy	self-reporting questionnaire	participants were requested to rate their degree of bitter, sour, salty, sweet or greasy (high-fat) food preference	ADRB2 (human beta 2-adrenergic receptor)	3

### Quality of included studies

The results of the quality assessment of studies using the NOS tool are shown in Supplementary Table 2. Among all the evaluated studies, 9 studies were of poor quality (score less than 5) [26, 29, 31, 37, 42, 51, 68–70] and the rest of the studies had good quality (overall score ≥ 5 points).

### Association between genes and food preference to macronutrients (carbohydrate, fat, and protein)

As shown in Table 2, in some studies, researchers have evaluated the relationship between genes and the preference to consume macronutrients. Prado-Lima et al., in a cross-sectional study, evaluated the association between the serotonin receptor 5-HT_2A_ gene and preference for micro and macronutrient intake. They found that participants with the TT genotype of the T102C polymorphism of the 5-HT_2A_ receptor gene had higher protein intake and a higher tendency toward beef compared with CC or TC subjects [26]. In a different study, Bauer et al. looked at the relationship between a few SNPs and macronutrient intake and discovered statistically significant relationships between five of the twelve SNPs that were situated in or close to the genes SH2B1, KCTD15, MTCH2, NEGR1, and BDNF and consumption of macronutrients. The risk allele at rs7498665 (SH2B1) was linked to higher intakes of total fat (1.08 g/d energy-adjusted; 95% CI: 0.36, 1.81), saturated fat (0.60 g/d; 95% CI: 0.22, 0.97), and monounsaturated fat (0.37 g/d; 95% CI: 0.04, 0.69). For the risk alleles of the SNPs in or close to KCTD15 and NEGR1, a reduction in monounsaturated fat intake was seen, but carriers of the risk allele for NEGR1 also had reduced intakes of saturated fat. Furthermore, individuals who carry this SNP in or near KCTD15 have been found to consume less fat and more mono- and disaccharides and total carbohydrates [30]. Also, It has been reported in a study by Han et al., that G protein-coupled receptor TAS1R1 and TAS1R3 polymorphisms were associated with macronutrient intake. They found that participants with CC alleles of the TAS1R3 rs307355 and rs35744813 consumed a higher amount of protein than T carriers. Additionally, people who had the TAS1R1 SNP rs34160967's GG genotype ingested more fat and calories than those who had the A genotype [51]. Researchers looked at the relationship between angiotensinogen (AGT) gene polymorphisms and food preferences in a study of the Japanese population and discovered that individuals with the MM/MT genotype of AGT Met235Thr in comparison to those with the TT genotype consumed more total lipids, cholesterol, and unsaturated free fatty acids. Also, they didn’t find a significant correlation between AGT polymorphism (rs7079) and the ACE I/D with food preference [71].

**Table 2:** Overview of genetic association studies related to food preference

Study	Evaluated Genes	SNPs	Food preferences	Main results
Ozawa, 2002 [25]	human leukocyte antigen (HLA) genes (DRB1, DQA1 and DQB1)	-	Preference and intake of cow’s milk	cow’s milk preference is negatively associated with the frequency ofHLA-DQA1*0102 allele
Mennella,2005 [69]	TAS2R38	A49P allele	Cereals with different sugar contents Coffee with different sugar contents	No correspondence between TAS2R38 genotypes and sweet preference
Prado-Lima, 2006 [26]	5-HT_2A_ receptor	T102C polymorphism	protein intake and frequency of animal food consumption	Subjects with TT genotype had higher protein intake and higher tendency toward beef comparing with CC or TC subjects
Keskitalo,2007 [9]	A locus on chromosome 16 (16p11.2)	-	pleasantness and the use frequency of 5 sweet foods (chocolate, candy, ice cream, sweet desserts, and sweet pastry)	Chromosome 16p11.2 may harbor genetic variations that affect the consumption of sweet foods
Mizuta, 2008 [27]	*LEP* *LEPR*	G□2548A (rs7799039) A19G (rs2167270)	Sweet food preference	The LEP A19G and LEPR R109K polymorphisms were associated with sweet preference
Bienertova-Vasku, 2008 [28]	leptin, leptin receptor, adiponectin, proopiomelanocortin and ghrelin genes	LEP –2548 G/A, LEPR Gln223Arg, POMC RsaI and AvaI, Arg51Gln and	percentage of protein, carbohydrates or fat or fiber in food	No associations of the examined polymorphisms with food preferences were observed
Bauer, 2009 [30]	FTO, MC4R, KCTD15, TMEM18, GNPDA2, SH2B1, MTCH2, NEGR1, ETV5 and BDNF	rs1121980 (FTO) rs17700633 (MC4R) rs17782313 (MC4R)	Protein intake, Fat intake, Carbohydrate intake	Five SNPs were associated with dietary intake and were in or near 5 loci: SH2B1 (particularly with increased fat), KCTD15 (particularly with carbohydrate intake), MTCH2, NEGR1, and BDNF
Bienertová-Vašků, 2010 [29]	leptin (LEP), LEP receptor (LEPR), adiponectin (ADIPOQ), IL-6 and pro-opiomelanocortin (POMC)	ADIPOQ rs2241766 ADIPOQ + 94T/G LEP rs2167270 LEPR rs1137101	percentage of dietary protein, carbohydrates and fat intake	None of the examined polymorphisms served as an independent predictor for percentage of daily energy intake from macronutrients
Ooi, 2010 [31]	TAS2R38	P49A	like or dislike of list of 36 mostly local Asian vegetables, 4 soy products and 37 sweet or fat foods	TAS2R38 P49A SNP is not a suitable predictor of body indices and food selection for the population
Hayes, 2011 [32]	TAS2R16 TAS2R3 TAS2R4	rs1308724 *TAS2R16* rs846672 *TAS2R16* rs765007 *TAS2R3*	Like/dislike of grapefruit juice and instant espresso	SNPs in TAS2R3, TAS2R4, and TAS2R5 formed a haploblock that explained coffee bitterness TAS2R19 variation influenced grapefruit juice bitterness and liking
Keller, 2012 [33]	CD36	rs1984112, rs1761667, rs1527483, rs1049673, and rs3840546	Acceptance and liking score of different fat containing food	rs1761667 genotype was associated with reported acceptance of added fats and oils
Pirastu, 2012 [34]	BDNF/BDNFOS, CD36, GNB3, GNG13, ITPR3	90 informative SNPs in 27 genes	Liking ratings of 20 common foods	There are significant associations between rs2277675 on the TRPV1 gene and liking for beet, rs28374389 on TAS1R2 gene with lamb meat liking, rs2290550 on PLCB2 gene and hot tea liking and non-wild type alleles of ITP3 gene variants (rs2229642 and rs3818521) with lower liking of lamb meat and sheep cheese
Eriksson, 2012 [36]	GWAS	rs72921001	cilantro preference	rs72921001 is associated with soapy-taste detection that is confirmed in the cilantro preference group. The C allele is associated with both detecting a soapy smell and disliking cilantro
Brunkwall, 2013 [35]	FTO	rs9939609	dietary intake from 27 food groups	A-allele carriers reported a higher consumption of biscuits and pastry but lower consumption of soft drinks compared to TT genotype carriers
ERGÜN, 2013 [37]	hTAS2R38	Rs713598	food choices	Polymorphisms on hTAS2R38 bitter taste receptor gene had no effect on food choices within the study population
Laaksone, 2013 [70]	hTAS2R38	A49P (rs713598), A262V (rs1726866), and V296I (rs10246939	The liking of odor, appearance, and flavor of Wild bilberries and Wild crowberries’ juice, extract and juice + extract	Based on the genotype grouping of subjects, PAV homozygotes gave lower ratings to the attributes than AVI homozygotes PAV homozygotes were predicted to dislike the extracts notably more than AVI homozygote
Sasaki, 2013 [71]	ACE, ADRB3 and AGT	AGT Met235Thr AGT rs7079 ACE I/D ADRB3 Trp64Arg	potato/sweet potato, beans, rice, bread, noodles/soba, fish/shellfish, small fish, meat, eggs, milk, dairy products, brightly collared vegetables	AGT Met235Thr gene polymorphism is linked to the food preferences of carbohydrates and total lipids, thereby contributing to an increase in energy intake
Wakai, 2013 [39]	ADIPOQ (adiponectin encoding gene)	rs822396	Confectionery-intake score	rs822396 SNP of ADIPOQ gene was correlated with a preference for confectionery
Pirastu, 2014 [40]	Twenty four TAS2R genes	88 SNPs	Coffee Liking	Two SNPs on the TAS2R43 gene (rs71443637 and rs35720106) were significantly associated with coffee liking
Törnwall, 2014 [41]	GWAS TAS1R1 PKD1L3	rs2235564 and rs6577584 rs12102451	Participants were classified into 2 groups (basic and adventurous) using clustering method, based on liking responses to food names representing sour, umami, and spicy flavor qualities	Linkage analysis for 27 candidate gene regions revealed suggestively that being adventurous is linked to TAS1R1 and PKD1L3 genes
Hayes, 2015 [42]	TAS2R19 TAS2R31	Arg299Cys (rs10772420) in TAS2R19 Val240Ile (rs10772423) and Ala227Val (rs10845293) in TAS2R31	liking of “unsweetened grapefruit juice.”	TAS2R19 Arg299Cys SNP is statistically associated with the bitterness of quinine and the liking of grapefruit juice individuals homozygous for Val240 reported a significantly greater mean liking for grapefruit juice than did either the or the Ile240 homozygotes
Robino, 2015 [43]	TAS1R2 GLUT2	rs3935570 rs1499821	sweet liking score	There was no association between studied SNPs and sugar intake
Wallace, 2015 [44]	COMT	rs 4680	food desirability and self-rated “healthy” and “unhealthy” food perceptions	individuals with the val/val and val/met, COMT genotype had greater desirability for objectively defined “unhealthy” food items, as compared to met/met individuals
Jayewardene, 2016 [45]	CD36	rs1527479 and rs1984112	self-reported fat preference	Fats and oil, as well as dairy consumption frequency, were not significantly different between genotypes at either SNP
Shen, 2016 [46]	hTAS2R38 CA6	Ala49Pro (rs713598), Val262Ala (rs1726866) and Ile296Val (rs10246939) (rs2274333)	rating of the bitter intensity perceived and liking of four vegetables	Regarding vegetable intake, a difference in total vegetable and brassica vegetable consumption was found between TAS2R38 genotypes, however, both PAV/PAV and AVI/AVI groups consumed more vegetables overall and more brassica vegetables than the PAV/AVI group. CA6 genotype did not show strong associations with vegetable intake
Pirastu, 2016 [21]	GWAS		Liking/disliking for 20 foods belonging to 4 different categories (vegetables, fatty, dairy and bitter)	15 independent genome-wide significant loci were associated with liking/disliking of 12 different foods
Deshaware, 2017 [47]	TAS2R38	rs713598, s1726866 and rs10246939	preferences of fruits, vegetables and dairy products	Food preferences did not significantly correlate with PROP or TAS2R38 status
Risso, 2017 [48]	TAS2R1 TAS2R4 TAS2R14	rs2234233 rs2234001 rs11610105, rs3741843, rs7138535 rs3935570, rs4073538, rs4920566	liking + consumption score of 12 common foods	A significant association was observed only between TAS2R38 SNPs and food preferences (vegetable liking + consumption score)
Shen, 2017 [49]	CD36 CA6	rs1761667 rs2274333	liking of ice cream and dietary fat intake	There was no association between CD36 or CA6 genotypes and liking for ice cream Participants with the rs2274333 A/A genotype of CA6, tending to have a lower intake of fat as a percentage of energy intake than the A/G genotype
Bartáková, 2018 [50]	TAS1R2 TAS2R7 TAS2R9 CD36 SLC2A2	(rs35874116) (rs619381) (rs3741845) (rs1527479) (rs5400)	Daily frequency of intake of eight categories of foods (Cereals, Vegetables, Fruit, Milk and dairy products, Protein food, Goodies (sweet and salty food), Sweet beverages, Alcoholic beverages	carriers of particular alleles or genotypes did not differ in the frequencies of particular food consumption categories
Han, 2018 [51]	TAS1R1 TAS1R3	rs41278020 rs34160967 rs35118458	total energy (kJ), carbohydrate, protein, fat or sweet foods); savoury foods	Participants identified with the CC alleles of the TAS1R3 rs307355 and rs35744813 consumed significantly more protein from the buffet than T carriers Participants with GG genotype of the TAS1R1 SNP rs34160967 consumed more fat and calories as compared to the genotype group having the A alleles
Lek, 2018 [52]	DRD2	Taq1A (rs1800497) Taq1B (rs1079597) Taq1D (rs1800498)	The preference/intake frequency/craving of 26 common high-fat Malaysian foods	Taq1A is associated with fast food preference Taq1B, particularly B1 allele, is also associated with preferred fast food more Taq1D, particularly D1 allele, is associated with increased starchy food craving and mamak food preference
Perna, 2018 [53]	TAS2R38	RS713598	Preference of 30 food items	polymorphism (RS713598) of the TAS2R38 gene does not influence food preferences (except for butter, beer and cured meat)
Eriksson, 2019 [54]	TAS1R1, TAS1R2, TAS1R3, TAS2R16, TAS2R38, TAS2R50, SLC2A2, SLC2A4, GNAT3, SCN1B and TRPV1	94 SNPS in the studied genes	The scores for foods representing the different tastes	Polymorphisms in the GNAT3, SLC2A4, TAS1R1 and TAS1R2 genes were associated with sweet food intake, variations in taste receptor, glucose transporter and gustducin encoding genes are related to taste perception, food preference and intake
Watanabe, 2019 [55]	ADRB3	Trp64Arg	participants were requested to rate their degree of bitter, sour, salty, sweet or greasy (high-fat) food preference	ADRB3 Trp64Arg (T/C) polymorphism has no significant impact in food preference of foods in four major taste groups however food preference for high-fat sweet foods in heterozygous group was significantly higher than that in wild-type group also this group significantly more like high-fat foods
Hwang, 2019 [56]	FTO TAS1R2 TAS1R3 GNAT3 GLUT2	rs11642841	Intake of total sugars Intake of sweets (biscuits (i.e.,cookies), chocolate, or sweets (i.e., candies))	a strong association was observed between the intake of total sugars and the single nucleotide polymorphism rs11642841 within the *FTO* gene on chromosome 16. However, no association was observed with TAS1R2, TAS1R3, GNAT3, and GLUT2
Pilic, 2020 [57]	SCNN1B TRPV1	rs239345 rs8065080	Dietary salt intake and preference of salty foods	rs8065080 had lower ratings of saltiness and higher ratings of pleasantness of soup
Kawafune, 2020 [14]	ALDH2	rs671	The taste preference for sweetness	There is an association of the rs671 variant which is located in the 12q24 locus with sweet taste preferences in Japanese populations
Park, 2020 [58]	TAS1R2 SLC2A5 SLC2A7 SLC2A5	rs61761364 rs11121306 rs769902	Preferences for each taste like sweet, salty, spicy, sour, and oily foods were asked	GRS was calculated by summing the number of sweet taste preference alleles of 8 genetic variants. a high GRS of 8 SNPs from TAS1R2, SLC2A5, SLC2A7, TRPM5, and TRPV1 had a positive association with sweet taste preference, compared to low GRS
Choi, 2021 [59]	CD36	rs1527479	carbohydrate foods, carbohydrate- and fat-rich foods, sweets, protein-rich foods	rs1527479 did not have a meaningful effect on the intake of fat or other macronutrients or on the selection of food among
Cornelis, 2021 [60]	GWAS		liking or consumption of coffee, tea and other bitter tasting foods; specifically, beer and dark chocolate	Variants near TMEM18, GCKR, POR, ADORA2A (rs2330783), CYP1A2 (rs2472297, rs762551), AHR, CYP2A6, SEC16B, OR5M7P, ENSA, and MLXIPL were significantly associated with total coffee intake variants near ABCG2, MC4R and AKAP6 were nominally associated with total coffee intake
Di´oszegi, 2021 [61]	TAS1R3 CD36 SCNN1B TRPV1 TAS2R38	rs307355 rs1761667 rs1527483 rs239345 rs8065080 rs713598	Rating of sweet-, fatty-, salty- and bitter-tasting food items	no associations were observed between certain genetic polymorphisms and taste and food preferences. CA6 rs2274333 with salty taste and raw kohlrabi preference, CD36 rs1527483 with fat taste preference, TAS2R19 rs10772420 with grapefruit preference, and TAS2R38 rs713598 with quantity of sugar added were related
Graham, 2021 [62]	CD36 TAS2R38	rs1761667 rs713598, rs1726866 rs10246939	total carbohydrate, total fat, monounsaturated fatty acid (MUFA), polyunsaturated fatty acid (PUFA), saturated fatty acid (SFA), and total protein were quantified	There was no association between either TAS2R38 diplotypes or CD36 rs1761667 and dietary intake There is a difference in SFA preference according to TAS2R38 rs1726866 and rs10246939 genotypes
Rana, 2021 [63]	MC4R BDNF FTO TMEM18 NEGR1	rs17782313 rs6265 rs1421085 rs7561317 rs2815752	Tendency toward fat-dense food	Only effect of interaction between studied gene variants and tendency toward fat-dense food on obesity related factors were reported and effect of studied gene variants on tendency toward fat-dense food was not reported
Suzuki, 2021 [64]	-	rs4982753	preference for a Japanese dietary pattern were assessed using Japanese food score	rs4982753, in the 14q11.2 locus was significantly associated with the Japanese food score The SNP, rs4982753 on the 14q11.2 locus did not hit any gene
Concas, 2022 [65]	CAV1 (caveolin 1)	Several SNPs	Liking of different foods and beverages	rs6961694 CAV1 SNP found to be also associated with liking of alcoholic beverages and of sweet foods
Fernández-Carrión, 2022 [66]	PTPRN2 (Protein Tyrosine Phosphatase Receptor Type N2)	rs2091718-PTPRN2	The preference for sugary foods, including “breakfast cereals”, “sweets-pastries and ice creams”, “chocolates” and “sugar”	sweet taste preference was strongly associated with sugary food liking several SNPs in the PTPRN2 gene (located at chromosome 7), significantly associated with sweet taste preference
May-Wilson, 2022 [67]	GWAS		Food-liking phenotypes	GWAS analysis identified 1,401 significant food-liking associations which showed substantial agreement in the direction of effects with 11 independent cohorts
Narita, 2022 [68]	ADRB2	Gly16Arg	participants were requested to rate their degree of bitter, sour, salty, sweet or greasy (high-fat) food preference	preference for sour food was significantly higher in the ADRB2 GG group than that in the ADRB2 CC group, but only for female subjects

However, in some studies, there was no meaningful connection between SNPs and macronutrient intake. Bienertova-Vasku et al. reported that there wasn’t any significant association between examined polymorphisms (LEP –2548 G/A, LEPR Gln223Arg, POMC RsaI and AvaI, Arg51Gln and Leu72Met in ghrelin gene, APM1 T94G) with abnormal eating patterns [28]. Also, rs1761667 G > A in the CD36 protein's genetic variant and the consumption of fat or other kinds of macronutrients or on the choice of food among non-obese males and females were not shown to be significantly correlated by Choi et al. [59]. In line with this study, Keller et al. demonstrated a relationship between the intake of extra fats and oils and the CD36 gene variant rs1761667 [33].

### Association between genes and food preference for sweet, salt and fatty foods

Mennella et al. examined the relationship between genetic variation in the TAS2R38 gene and food preference in a cross-sectional study, and they discovered that genotypes at the TAS2R38 locus substantially linked with a higher liking for sucrose and sweet-tasting meals and beverages, including cereals [69]. In line with this finding, Keskitalo et al. reported a significant association between a locus on chromosome 16 and a preference for 5 sweet foods (chocolate, candy, ice cream, sweet desserts, and sweet pastry) [9]. Another study looked into the relationship between the leptin gene (LEP) and leptin receptor gene (LEPR) polymorphisms and food preferences. It found that the LEP A19G and LEPR R109K polymorphisms are connected to a desire for sweet foods [28]. Also, Kawafune et al. examined the association between the 12q24 locus and sweet taste preference in the Japanese population and found a significant correlation [14]. In a population-based study among Korean adults, the researchers evaluated the association between genetic risk scores(GRS) which contained 8 SNPs (TAS1R2_rs61761364, SLC2A5_rs11121306, SLC2A7_ rs769902, SLC2A5_rs765618,

TRPM5_rs1965606, TRPV1_rs224495, TRPV1_ rs8065080, and TRPV1_rs8078502) and sweet taste preference and they found a 1.30-folds increase in GRS was associated with higher sweet taste preference [58]. Moreover, in another study on Hungarian general and Roma populations, researchers looked at the relationships between taste and dietary preferences and the polymorphisms TAS1R3, CD36, SCNN1B, TRPV1, TAS2R38, TAS2R19, and CA6. The findings revealed a significant association between CA6 rs2274333 and a preference for raw kohlrabi and salt, CD36 rs1527483 and a preference for fat, TAS2R19 rs10772420 and a preference for grapefruit, and TAS2R38 rs713598 and a preference for the amount of sugar added [47].

In some studies, it has been reported that the adiponectin encoding gene (ADIPOQ gene) especially the rs822396 SNP, is related to confectionery intake [39]. Another study didn’t find a significant correlation between TAS1R2 and GLUT2-related SNPs with sweet liking scores [43].

### Association between genes and food groups' preferences

In some studies, researchers evaluated the effects of genetic variation on food groups’ preferences. Ozawa et al. in 2002 evaluated the association between genetic variation in human leukocyte antigen (HLA) genes (DRB1, DQA1, and DQB1) and cows' milk preference and discovered a negative correlation between the prevalence of the HLA-DQA1*0102 allele and liking for cows milk [25]. In another population-based study among subjects from the Caucasus and Central Asia, in 2012, the preference for certain food items was examined by scientists who assessed the relationship between genetic variations with the TAS1R2, TAS1R2, PCLB2, TPRV1, and ITPR3. It has been shown that there are significant correlations between TAS1R2 and TAS1R3 variants and liking Vodka, white wine, and lamb meat, PCLB2 gene and preference for Hot Tea, TPRV1 gene and liking of beet, and ITPR3 gene and liking of both lamb meat and sheep cheese [34]. Another study conducted by Brunkwall et al. investigated how variations in the fat mass and obesity-associated gene (FTO) are linked to dietary preferences in individuals without any health issues. They found that A-allele carriers reported a higher intake of some energy-dense foods such as biscuits and pastries but lower consumption of soft drinks in comparison with TT allele carriers [35].

In a cross-sectional study, the investigators didn’t find any significant correlation between the bitter taste receptor gene hTAS2R38 and food choices [37]. Also, there wasn’t any significant correlation between CD36 protein SNPs (rs1527479 and rs1984112) with fats and oil, as well as dairy consumption frequencies [45].

Furthermore, there have been investigations into the association between genetic variations and the level of craving for unhealthy foods. Wallace et al. conducted a cross-sectional study to explore the link between the dopamine-related catechol-O-methyltransferase (COMT) gene and the appeal of "unhealthy" foods. The study discovered that individuals with Val/Val and Val/Met genotypes of the COMT gene showed a higher desire for objectively identified "unhealthy" food items compared to those with the Met/Met genotype [44].

### The correlation between genetic variations and the inclination to consume bitter-tasting foods

Multiple studies have examined the connection between genetic variations and individuals' inclination towards consuming vegetables. Shen et al. conducted a cross-sectional study to investigate the connection between TAS2R38 and gustin (CA6) gene variations and their correlation with a preference for brassica vegetables. The study revealed that individuals possessing the TAS2R38 AVI/AVI genotype exhibited a greater preference for brassica vegetables. Also, they found that both PAV/PAV and AVI/AVI subjects consumed more total vegetables and brassica vegetables than PAV/AVI cariers [46]. Pirastu et al. in a population-based study among an Italian population evaluated the Genome-Wide Association (GWAS) with common food likings and reported that seven loci were associated with vegetables. They found that some SNPs such as rs10050951, rs8034691 and rs28849980 were associated with artichokes liking. Also, they found two loci for broccoli liking (rs2530184 located in a gene desert region on chromosome 17 and rs9832668 located on chromosome 3 close to the RYBP gene), and finally one locus on chromosome 8, very close to the CSMD1 gene (rs138369603) which was associated with chicory liking. Moreover, in terms of bitter foods, they found 3 loci, one for dark chocolate (rs73082019), one for coffee (rs145671205), and one for liver liking (rs34088951) [21]. Risso et al. evaluated 183 volunteers from four geo-linguistic groups and found a significant correlation between rs860170 (TAS2R16) and the desire to consume bitter vegetables including broccoli, mustard, and beer [48]. In another study, Perna et al. found that RS713598 SNP of the TAS2R38 gene was associated with a higher preference for beer [53]. Similar findings were reported between caveolin 1-related SNPs and liking alcoholic beverages [65]. In another study, Hayes et al. showed that SNPs in TAS2R3, TAS2R4, and TAS2R5 are significantly correlated with the desire to consume bitter coffee and alcohol intake [32]. Moreover, a significant association was shown between TAS2R gene-related SNPs and coffee liking [40].

However, the results of some studies were contradictory. Deshaware et al. in a study among Indian subjects didn’t find any significant correlation between bitter taste receptor gene TAS2R38 polymorphisms and food preference for vegetable or bitter foods [47].

## Discussion

This study is the initial systematic review conducted among adults, with the aim of analyzing how genetic variation influences food preferences. Dietary behavior in people is influenced by various factors, and one of the most significant factors is genetics [72]. Food preference take form in the course of fetal development, and eating habits undergo changes as time progresses. This intricate characteristic is influenced by a combination of genetic and environmental elements. The sensory attributes of ingested food play a crucial role in shaping dietary habits, with taste recognized as a primary influencer of food choices and dietary patterns [3]. Chemical compounds present in food trigger specialized taste receptors, and these receptors can be influenced by genetic variations, resulting in individual variations in taste and preferences. The perception of bitter, sweet, and umami is associated with G-protein-coupled receptors [73, 74], while salt and sour tastes are governed by ion channels [75]. In this regard, SNPs in taste receptor genes are among the most studied polymorphisms [76]. For instance, sugar consumption in humans has been linked to sweet taste receptor (TAS1R2) alleles [77, 78]. Additionally, the consumption of vegetables, oil, and sweets has been associated with the genetic variation of the bitter taste receptor TASR38 [79].

The effect of TAS1R and TAS2R gene families on dietary behavior, specifically the preference for sweet and bitter tastes, has been investigated in the majority of studies examining the relationship between genetic variants and dietary behavior. Monosodium glutamate (MSG) is commonly used in humans to stimulate the heterodimeric G protein-coupled receptors TAS1R1 and TAS1R3 to perceive umami tastes [74, 80]. There are three proteins in the TAS1R family receptors, TAS1R1, TAS1R2, and TAS1R3, encoded by their respective genes, TAS1R1, TAS1R2, and TAS1R3. It has been reported that TAS1R2 and TAS1R3 create a heterodimer, and this heterodimer through the connections and effects on specific receptors, lead to responds to sweet tastes, including sugars, artificial sugars, d-amino acids, and some proteins, such as miraculin [81, 82]. Previous studies have shown that individuals with the GG genotype of the TAS1R1 SNP rs34160967 and the CC genotype of the TAS1R3 SNP rs307377 had lower levels of MSG threshold [83]. Furthermore, certain studies have indicated a notable association between the TAS1R2 gene and an increased preference for vodka and white wine [34, 84]. Indeed, research has proven that alcohol and sucrose activate the identical gustatory neural pathway [85]. Brasser et al. in an animal study found that in knockout mouse models for the TAS1R3 gene, the preference for alcohol and the amount of alcohol consumption significantly reduced [86]. In line with these findings, Hinrichs et al. found that TAS2R16 has an impact on alcohol liking [87].

Additional research has examined how variations in genes related to obesity and adipose tissue impact individuals' food preferences. Among these genes, the fat mass and obesity-associated gene (FTO) holds particular significance. Brunkwall et al. demonstrated that individuals with the FTO A-allele may not only display a greater appetite overall but also exhibit a preference for certain food categories. Specifically, they showed a tendency for higher consumption of biscuits, cereals, high-fat meat, and pastry, while having a lesser preference for soft drinks compared to those with the TT allele [35]. Also, it has been reported in one study among children that the A-allele carriers consume higher amounts of energy-dense foods. In a study by Hwang et al. on the UK Biobank sample, a significant association between the rs11642841 variant of FTO and total sugar intake was reported [56]. These results suggest that some variants of FTO can increase the risk of obesity by increasing the tendency to consume sweet and high-calorie foods.

The findings from studies examining the relationship between genes and the preference for fat taste and fat intake are inconsistent. It is believed that the CD36 gene, responsible for producing the fatty acid translocase, plays a role in detecting fatty acids in the mouth. This protein acts as a scavenger receptor and facilitates the transfer of long-chain fatty acids into cell membranes, which is crucial in the breakdown of fats. Considerable research focus has been directed toward the analysis of fat taste perception in relation to two SNPs, namely rs1761667 and rs1527483, situated within the CD36 gene [88]. The CD36 gene encodes the fatty acid translocase, a crucial element in the initial step of fat metabolism, responsible for transporting long-chain fatty acids (LCFA) across cell membranes. Some studies indicated that individuals with the AA genotype of rs1761667 in the CD36 gene had higher thresholds for perceiving lipid taste compared to those with the GG genotype [33, 89]. In a particular study conducted on the UK population, no notable association was observed between TAS2R38 diplotypes or CD36 rs1761667 and the consumption of dietary fat. However, researchers did discover a meaningful relationship between the sensitivity to bitter taste and the intake of saturated fatty acids [62]. Another genetic variant that has been associated with the tendency to consume oil and fatty foods is rs6661761 located within the BPNT1. Although precise data about BPNT1 gene function is not available, It is highly expressed throughout the brain and is severely suppressed by lithium, a medication that is frequently used to treat bipolar disorder. In rats conditioned to anhedonic responses, lithium has been demonstrated to recover hedonic responses to appetizing stimuli through the nucleus accumbens [90]. Some researchers suggest that liking oil and fatty foods might be linked to the reward of palatable foods through the nucleus accumbens [21]. There is a linear negative association between the function of this nucleus and obesity risk. So, BPNT1 is known as a good indicator for understanding the physiology underlying the liking of palatable foods and the activation of the reward system [91].

Based on our knowledge, this study is the first systematic review that evaluates the association between genetic variants and food preference. Our study had several strengths, including conducting a grey literature search, no language restrictions in the screening of studies, and also examining a wide range of genetic variants. However, this study had limitations that should be considered in the interpretation of the results. Although the aim of this study was to perform a meta-analysis, due to the high heterogeneity between the examined variants and the impossibility of pooling the data, this was not possible, and the study was written in a systematic review form. On the other hand, the tools used in the primary studies to evaluate dietary tendencies were diverse (some studies used self-filled questionnaires, some online questionnaires, interviews, and other methods), which affects the accuracy of the results.

## Conclusion

In conclusion, this systematic review study showed that there was a considerable association between some of the genetic variants with food preferences among adults. Some of these genetic variants increase or decrease the desire for sweet and fatty foods, and some affect the choice of food groups. However, due to the high heterogeneity in the investigated variants, more studies are needed to investigate these genetic variants more closely and to identify the mechanisms involved in the observed effects.

### Supplementary Information

**Additional file 1. **

## Data Availability

The data produced or examined in this study are incorporated in this article and can be obtained from the corresponding author upon reasonable inquiry.
